# Comprehensive sequence and expression profile analysis of the phosphate transporter gene family in soybean

**DOI:** 10.1038/s41598-022-25378-w

**Published:** 2022-12-03

**Authors:** Xiaoshuang Wei, Yu Fu, Renjie Yu, Lei Wu, Zhihai Wu, Ping Tian, Siyuan Li, Xue Yang, Meiying Yang

**Affiliations:** 1 College of Agronomy, Jilin Agricultural University, Changchun, 130118 Jilin China; 2 College of Life Sciences, Jilin Agricultural University, Changchun, 130118 Jilin China; 3 National Crop Variety Approval and Characterization Station, Jilin Agricultural University, Changchun, 130118 Jilin China

**Keywords:** Biological techniques, Molecular biology

## Abstract

The family of phosphate transporters (PHTs) mediates the uptake and translocation of Pi inside the plants. However, little is known about transporters in soybean. Therefore, Searched the Genome Database for Soybean, 57 *GmPHTs* family members were identified in soybean, Phylogenetic analysis suggested that members of the PHTs gene family can be divided into six clades. Collinearity analysis revealed that most of the *GmPHT* genes shared syntenic relationships with PHTs members in *Arabidopsis thaliana* and that large segment duplication played a major driving force for *GmPHTs* evolution in addition to tandem duplication. Further analysis of the promoter revealed that light-responsive elements and abiotic stress-responsive elements were widely distributed within the promoter regions of *GmPHT* genes. Based on RNA-seq data, *GmPHTs* showed different expression patterns in roots and leaves of soybean treated with long-term low phosphorus and short-term low phosphorus, in addition, the expression levels of *GmPHT* genes can be regulated by drought stresses, it was implied that the induced expression of *GmPHTs* could promote phosphorus uptake and transport in soybean and thus adapt to low phosphorus and drought stress, which is the first step dissection of Pi transport system and probably refers to new roles of PHTs genes in soybean.

## Introduction

Phosphorus (Pi) is a key nutrient for plant growth and development, is the second essential macronutrient required for plant growth and development alongside nitrogen^1^ and is also an essential component of fertilizers used to sustain modern agriculture because it helps quality and yield. Pi is part of important biomolecules and, in the form of phosphate, pyrophosphate, adenosine triphosphate, adenosine diphosphate, or adenosine monophosphate, involved in various metabolic functions and many living cell regulatory processes are P-dependent^2^. Globally, many soils are deficient in phosphate (Pi)^3^, Pi concentrations within plant cells are typically 1000-times those outside^4^. Therefore, plants must have specialized transport vehicles to transfer Pi from the soil to the cells to counter the large concentration gradient at the root-soil interface. The active phosphorus in the soil and the phosphorus used in various metabolic processes ultimately require direct uptake and transport through the "phosphate transporter protein (PHT)", a highly efficient plant root uptake and transport system, which is responsible for the transport of inorganic phosphorus in plants under the regulation of transcription factors. Genome sequence analyses and experimental evidence have indicated that plants contain numerous Pi transporter families, including *PHT1, PHT2, PHT3, PHT4,* and *PHT5*, which were distinguished by their protein sequences, structures, locations, and functions^5–11^.

The *PHT1* family has been most extensively studied in plants, and the main pathway for Pi to enter plants from the soil is through the PHT1 family located on the plasma membrane, PHT type 1 protein is also defined as a major contributor to the Pi uptake system^12^ and the plant has several PHT1 transporter members that play a key role in P acquisition, transport and remobilization. The *Arabidopsis AtPHT1* gene family contains nine members, The rice *OsPHT1* gene family has 13 members^13^. The first high H + /PI phosphate transporter identified in higher plants was AtPT1 from *Arabidopsis*^14^. This gene has an important role in the uptake of phosphorus from the soil^15^. Analysis of expressed sequence tags (ESTs) and genomic sequences revealed that nine genes in *Arabidopsis* are similar to *AtPT1*, with *PHT1;1* transcript being the most abundant^16^. Its overexpression increases Pi uptake in *Arabidopsis*^17^. The *PHT2* gene family contains one member each in *Arabidopsis thaliana* and rice^13^, and orthologous gene have been characterized in wheat, alfalfa rhizobia and several Solanum species^14^. Arabidopsis PHT2;1 is the first member of the PHT2 family to be identified, with OsPHT2;1 in rice being the putative low-affinity phosphate transporter gene involved in the accumulation and transport of Pi in the plant^18^. The third family of plant Pi transporters is localized to the mitochondria, Three *PHT3* genes have been identified in Arabidopsis^8^ and six members identified in rice^13^, and homologs have been cloned and partially characterized soybean, maize, and birch^19,20^. The Arabidopsis genome contains six *PHT4* genes (*PHT4;1* to *PHT4;6*), and chloroplast localization of *PHT4;1, PHT4;2, PHT4;4*, and *PHT4;5* has been analyzed using GFP fusions or immunoblotting^21–23^. In Arabidopsis, phosphate transporter proteins containing the SPX–MFS structural domain have been designated as members of the phosphate transporter protein family *PHT5* or VPT. The results also confirmed that *PHT5* could increase plant tolerance under low phosphorus stress. The results also confirmed that *PHT5* could increase plant tolerance under low phosphorus stress^24^. *GmPHT5* in soybean may be responsible for transporting phosphorus from root vascular tissues to nodules, especially under phosphorus deficiency stress, and therefore *GmPT5* may be a key transporter in regulating nodule phosphorus homeostasis^25^.

Soybean (*Glycine max* (L.) Merr.) is an economical and agronomical crop, which has been adopted as a diet staple throughout many parts of the world^6^, However, the low availability of phosphorus (P) in the soil is the main nutrient limiting factor for soybean yield^26,27^. Previous studies have reported that phosphorus addition improves drought tolerance in soybean^28,29^. Phosphorus application increased nitrogen fixation rates in soybean^30,31^ which is sensitive to drought stress^32–34^. In production areas within China, drought is the most challenging stress for soybean^35^ and the non-renewable resource of P is predicted to have limited availability, soybean seed yield will be threatened by both water and P deficits in the future^36^. Therefore, it is urgent that researchers develop plants with enhanced efficiency of soil phosphorus use under such conditions, improve the capacity of soybean to absorb phosphorus. Because Pi is moved from the soil into plant cells in response to excess phosphate, genomic analyses have been conducted with Pi transporter families in Arabidopsis, rice and apple.

Currently, the study of phosphorus transport protein genes in Arabidopsis, rice and other plants has gained great progress, while PHTs have been rarely studied in soybean. Therefore, based on the published *Arabidopsis thaliana* genome, this study identified and characterized putative soybean PHTs family genes through phylogenetic analysis, collinearity analysis, motif, promoter element analysis, and investigated the expression profile of PHTs genes in response to low phosphorus and drought. Transcriptome sequencing was used to analyze the induction of expression of root and leaf phosphorus transporter-related genes in soybean by low phosphorus treatment. The objective was to present a foundation for further functional dissection of *GmPHTs* so that genetic engineering approaches can be applied to improve the efficiency of phosphate uptake by stressed soybean plants.

## Result

### Identification of the PHTs family genes in soybean

Based on the homology with PHTs family members in *Arabidopsis,* a total of 57 PHTs genes were identified in the whole genome of soybean (Table 1). Each subfamily member also has its own characteristics. The differences in the isoelectric point and hydrophilicity of the subfamily proteins are relatively obvious, Except for the GmPHT5 subfamily, the isoelectric points of all subfamily members are basic, the isoelectric point of the *GmPHT5* subfamily is acidic, at around 6. The *GmPHT1, GmPHT2, GmPHT3, GmPHT4,* and *GmPHT5* subfamilies had positive hydrophilicity values ranging from 0.101 to 0.659, while the *GmPHO1* subfamily had negative hydrophilicity values ranging from − 0.255 to 0.064. In contrast, the hydrophilicity among the members of the *GmPHT4* subfamily varied considerably, with GRAVY values ranging from 0.2 to 0.6. The differences in the basic physicochemical properties of the proteins reflect the differences in their physiological functions. In particular, the differences in isoelectric point and hydrophilicity exhibited among the members within the GmPHT4 subfamily herald the diverse functional differentiation among the members of the GmPHT4 subfamily.

**Table 1:** Localization and physicochemical properties of members of the *GmPHTs* protein gene family.

												Predicted subcellular location	
Name	Gene locus^1^	Gene identifier^2^	Chr	Locations	Length(bp)	Protein ID^1^	Length(aa)	*p*I	Mw(kDa)	GRAVY	TMHs	WoLF PSORT	Softberry
*GmPHT1.1*	LOC100780201	Glyma.02G005800	2	634,487..639193	1602	NP_001241164.1	533	8.31	58.5	0.309	12	vacu:7	Plasma membrane
*GmPHT1.2*	LOC100819445	Glyma.03G162800	3	37,772,793..37775073	1620	NP_001304639.2	539	8.67	59.3	0.291	11	plas:10	Plasma membrane
*GmPHT1.3*	LOC100797683	Glyma.07G222700	7	39,866,989..39873025	1536	NP_001241574.1	511	8.51	57.6	0.295	12	plas:11	Plasma membrane
*GmPHT1.4*	LOC100795623	Glyma.10G006700	10	662,561..665361	1602	NP_001341396.1	533	8.54	58.4	0.324	12	plas:7	Plasma membrane
*GmPHT1.5*	LOC100786638	Glyma.10G036800	10	3,233,051..3236286	1566	NP_001304588.2	521	8.63	57.3	0.363	11	plas:7	Plasma membrane
*GmPHT1.6*	LOC100802365	Glyma.10G186400	10	41,935,063..41939261	1584	NP_001239971.1	527	8.91	58.1	0.397	11	plas:9	Plasma membrane
*GmPHT1.7*	LOC100802890	Glyma.10G186500	10	41,946,209..41950274	1611	NP_001240032.1	536	8.34	58.7	0.334	11	plas:10	Plasma membrane
*GmPHT1.8*	LOC100802261	Glyma.13G040200	13	12,579,481..12581070	1590	NP_001241400.1	529	8.82	58.7	0.316	10	plas:12	Plasma membrane
*GmPHT1.9*	LOC100805284	Glyma.14G123500	14	18,527,015..18528592	1578	NP_001241127.1	525	8.33	58.2	0.330	11	plas:11	Plasma membrane
*GmPHT1.10*	LOC100820250	Glyma.14G188000	14	45,277,372..45278961	1590	NP_001239765.1	557	8.18	61.2	0.232	12	plas:12	Plasma membrane
*GmPHT1.11*	LOC100803626	Glyma.19G164300	19	42,521,537..42524774	1620	NP_001254802.1	539	8.52	59.3	0.291	11	plas:10	Plasma membrane
*GmPHT1.12*	LOC100803834	Glyma.20G021600	20	2,196,534..2205616	1521	NP_001345390.1	506	8.63	56.8	0.333	11	plas:10	Plasma membrane
*GmPHT1.13*	LOC100792177	Glyma.20G204000	20	44,094,584..44098582	1611	NP_001240239.1	536	7.63	58.6	0.341	12	plas:11	Plasma membrane
*GmPHT1.14*	LOC100792711	Glyma.20G204100	20	44,103,550..44107427	1584	NP_001240957.1	527	8.93	58.1	0.386	11	plas:9	Plasma membrane
*GmPHT2.1*	LOC100787472	Glyma.08G282100	8	38,707,723..38713794	1728	XP_003530627.1	575	9.28	60.7	0.519	12	plas:7	Plasma membrane
*GmPHT2.2*	LOC100787349	Glyma.18G144100	18	23,306,379..23312679	1731	XP_003551229.1	576	9.31	60.5	0.502	12	plas:7	Plasma membrane
*GmPHT3.1*	LOC100788561	Glyma.01G157100	1	49,479,697..49484208	921	XP_003517120.1	306	9.13	33.8	0.167	1	chlo:5	Mitochondrial
*GmPHT3.2*	–	Glyma.02G065800	2	5,845,126..5847769	873	–	347	8.69	37.8	0.105	0	vacu:5	Mitochondrial
*GmPHT3.3*	LOC100791467	Glyma.05G201400	5	38,507,610..38510859	1080	XP_003524479.2	359	9.50	38.3	0.135	0	chlo:9	Mitochondrial
*GmPHT3.4*	LOC100800530	Glyma.08G008900	8	693,228..696708	1068	XP_003532391.2	355	9.37	38.0	0.115	0	chlo:9	Mitochondrial
*GmPHT3.5*	LOC100788974	Glyma.11G087800	11	6,610,115..6614787	921	XP_003537697.1	306	9.22	33.7	0.186	1	nucl:5	Mitochondrial
*GmPHT3.6*	LOC547633	Glyma.16G050000	16	4,788,380..4801207	1119	NP_001235652.2	372	9.25	39.8	0.163	0	chlo:4	Mitochondrial
*GmPHT3.7*	LOC100801963	Glyma.16G050100	16	4,798,523..4800531	1020	XP_025981816.1	339	9.38	36.8	0.184	0	chlo:11	Mitochondrial
*GmPHT3.8*	LOC100804257	Glyma.16G146700	16	30,755,899..30759360	1050	XP_003548029.1	350	9.25	37.6	0.101	0	cyto:8	Mitochondrial
*GmPHT3.9*	LOC548006	Glyma.19G101100	19	34,833,393..34841869	1128	NP_001237304.1	375	9.35	39.8	0.193	0	chlo:4	Mitochondrial
*GmPHT4.1*	LOC100789297	Glyma.02G224200	2	41,175,313..41180091	1551	XP_003519242.1	516	10.01	55.7	0.479	12	plas:7	Membrane bound chloroplast
*GmPHT4.2*	LOC100782221	Glyma.03G008200	3	793,191..799880	1782	XP_003520968.1	593	9.36	65.5	0.220	10	plas:11	Membrane bound chloroplast
*GmPHT4.3*	LOC100793618	Glyma.07G069600	7	6,307,073..6313498	1779	XP_003528848.1	592	9.37	65.2	0.218	10	plas:10	Membrane bound chloroplast
*GmPHT4.4*	LOC100819182	Glyma.07G144700	7	17,231,556..17235971	1515	XP_003529140.1	504	9.88	55.0	0.509	11	chlo:6	Membrane bound chloroplast
*GmPHT4.5*	LOC100812608	Glyma.07G274200	7	44,592,193..44600634	1290	NP_001239989.1	429	9.58	46.6	0.659	10	plas:8	Membrane bound Golgi
*GmPHT4.6*	LOC100787020	Glyma.11G175800	11	20,331,840..20340143	1554	XP_006591210.1	517	9.41	57.3	0.223	10	plas:9	Membrane bound chloroplast
*GmPHT4.7*	LOC100806856	Glyma.13G162900	13	27,823,310..27833361	1587	XP_003541488.1	525	7.16	57.4	0.339	7	chlo:7	Membrane bound chloroplast
*GmPHT4.8*	LOC100785142	Glyma.14G190900	14	45,569,552..45574890	1548	XP_003544870.1	515	9.93	55.8	0.540	11	plas:7	Membrane bound chloroplast
*GmPHT4.9*	LOC100809973	Glyma.17G000300	17	17,407..21108	1290	XP_003550165.1	429	9.68	46.5	0.624	10	plas:8	Membrane bound Golgi
*GmPHT4.10*	LOC100792616	Glyma.17G108300	17	8,486,013..8495685	1575	XP_003549724.1	524	6.83	57.5	0.330	7	plas:7	Membrane bound chloroplast
*GmPHT4.11*	LOC100792104	Glyma.18G066000	18	6,041,786..6050082	1476	XP_003551308.1	491	9.09	53.8	0.411	10	plas:6	Membrane bound chloroplast
*GmPHT4.12*	LOC100784401	Glyma.20G002000	20	213,184..219283	1794	XP_003556597.1	597	9.12	65.7	0.295	10	plas:10	Membrane bound chloroplast
*GmPHT5.1*	LOC100808181	Glyma.09G128500	9	32,046,109..32052575	2088	XP_003533972.1	695	6.17	78.2	0.214	11	plas:10	Membrane bound vacuolar
*GmPHT5.2*	LOC100777530	Glyma.09G263400	9	48,091,634..48104775	2094	XP_003534584.1	697	6.14	78.6	0.209	10	plas:11	Membrane bound vacuolar
*GmPHT5.3*	LOC100777032	Glyma.10G229900	10	46,001,071..46006711	2094	XP_014618833.1	697	6.22	78.7	0.170	11	plas:11	Membrane bound vacuolar
*GmPHT5.4*	LOC100778938	Glyma.16G176100	16	33,736,100..33743654	2088	XP_003548146.1	695	6.36	78.2	0.200	11	plas:10	Membrane bound vacuolar
*GmPHT5.5*	LOC100793709	Glyma.18G228700	18	51,774,933..51789151	2094	XP_006602776.1	697	6.55	78.7	0.232	11	plas:12	Membrane bound vacuolar
*GmPHT5.6*	LOC100805436	Glyma.20G163400	20	40,093,249..40098816	2094	XP_003556130.1	697	6.52	78.9	0.158	11	plas:11	Membrane bound vacuolar
*GmPHO1.1*	LOC100786083	Glyma.01G091800	1	27,867,550..27874181	2376	XP_003516868.1	791	9.14	92.2	− 0.212	6	plas:8	Plasma membrane
*GmPHO1.2*	LOC100796500	Glyma.02G003700	2	431,852..445384	2292	XP_006574509.1	763	9.24	88.4	− 0.106	6	plas:10	Plasma membrane
*GmPHO1.3*	LOC100795093	Glyma.02G110600	2	10,670,899..10676578	2256	XP_006574913.1	751	8.85	88.5	− 0.316	4	plas:8.5	Plasma membrane
*GmPHO1.4*	LOC100787164	Glyma.02G130200	2	13,340,016..13346701	2370	XP_003518826.1	789	9.12	92.0	− 0.219	6	plas:8	Plasma membrane
*GmPHO1.5*	LOC100819185	Glyma.07G228400	7	40,691,412..40698177	2412	XP_006583960.1	803	9.45	93.4	− 0.183	9	plas:12	Plasma membrane
*GmPHO1.6*	LOC100780548	Glyma.09G235200	9	45,765,397..45771516	2280	XP_006587738.1	759	9.28	87.7	− 0.103	6	plas:11	Plasma membrane
*GmPHO1.7*	LOC100781658	Glyma.10G004800	10	457,173..468529	2295	XP_006588543.1	764	9.41	88.4	− 0.111	5	plas:9	Plasma membrane
*GmPHO1.8*	LOC100794580	Glyma.10G183300	10	41,633,437..41645279	2325	XP_006589283.1	774	9.06	90.1	− 0.117	6	plas:10	Plasma membrane
*GmPHO1.9*	LOC100795104	Glyma.18G261900	18	54,775,710..54781535	2331	XP_003552542.1	776	9.24	89.3	− 0.114	6	plas:9	Plasma membrane
*GmPHO1.10*	LOC100805979	Glyma.20G031700	20	3,929,374..3936931	2409	XP_006605562.1	802	9.27	93.0	− 0.163	8	plas:12	Plasma membrane
*GmPHO1.11*	LOC100811310	Glyma.20G032400	20	4,183,317..4191520	2388	XP_003556776.1	795	9.38	92.8	− 0.233	8	plas:12	Plasma membrane
*GmPHO1.12*	LOC100813281	Glyma.20G032500	20	4,194,291..4202575	2391	XP_003556778.1	796	9.32	92.6	− 0.195	8	plas:11	Plasma membrane
*GmPHO1.13*	LOC100813818	Glyma.20G032600	20	4,204,879..4212818	2361	XP_003556779.1	786	9.24	91.7	− 0.121	8	plas:12	Plasma membrane
*GmPHO1.14*	LOC100802251	Glyma.20G206900	20	44,375,656..44385721	2325	XP_006606372.1	774	9.14	89.9	− 0.122	8	plas:9	Plasma membrane

Indicates that the corresponding PHTs gene was not found in the NCBI soybean database.

1 represents the number of the GmPHT gene in the NCBI soybean database.

2 represents the number of the GmPHT gene in the Phytozome soybean database.

### Phylogenetic analysis of the GmPHTs genes

**Figure 1:**
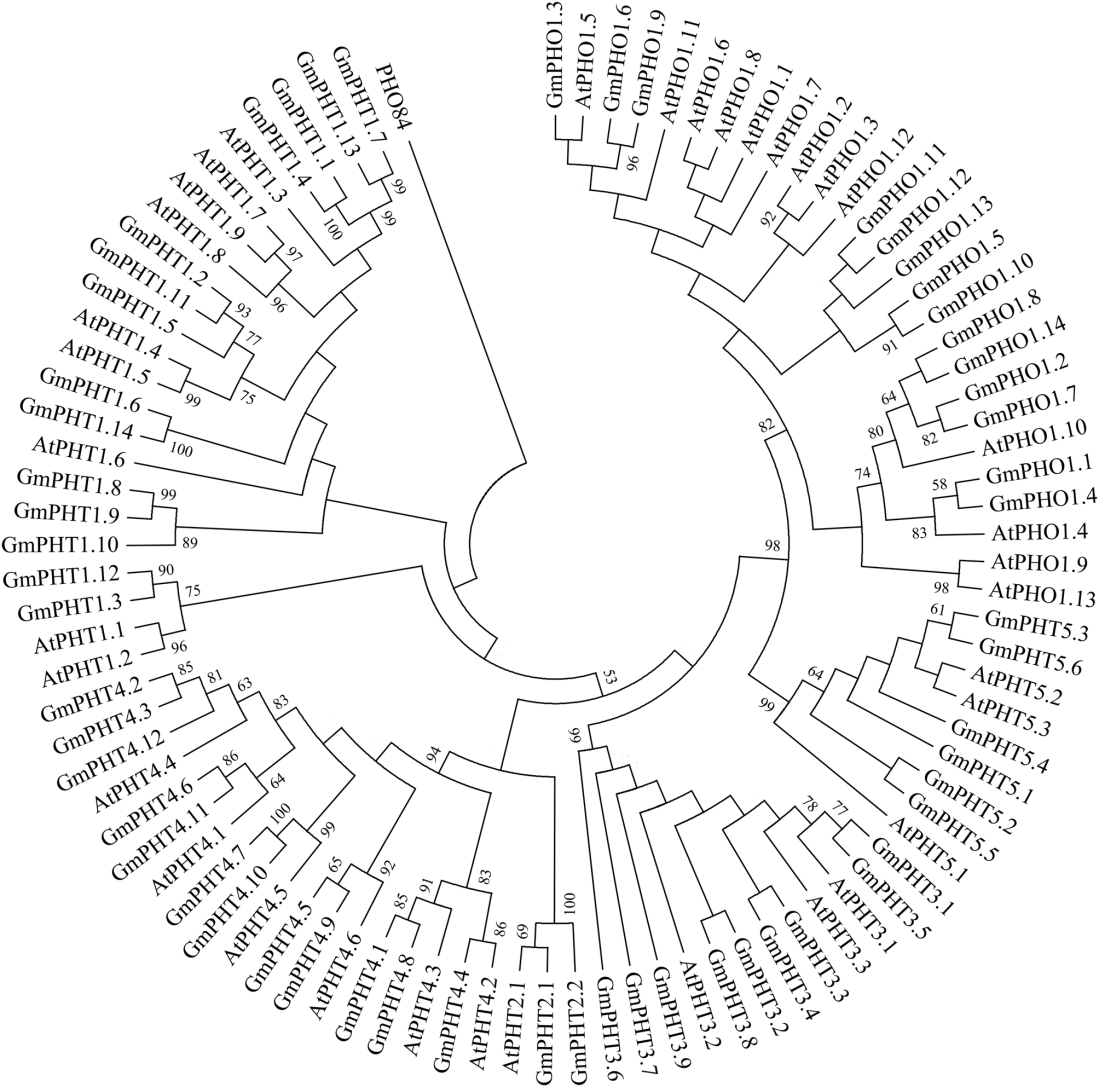
Phylogenetic tree of coding nucleotide sequences of the phosphate transporter family (PHT) in soybean and *Arabidopsis thaliana*. *Note*: The names of the genes used in soybean are referred to Table 1, and the names of the genes used in *Arabidopsis* are as follows. At1g68740(AtPHO1.1), At2g03260(AtPHO1.2), At1g14040(AtPHO1.3), At4g25350(AtPHO1.4), At2g03240(AtPHO1.5), AT2G03240(AtPHO1.6), At1g26730(AtPHO1.7), At1g35350(AtPHO1.8), At3g29060(AtPHO1.9), At1g69480(AtPHO1.10), AT3G29060(AtPHO1.11), AT4G25350(AtPHO1.12), AT5G35730(AtPHO1.13), AT5G43350(AtPHT1;1), AT5G43370(AtPHT1;2), AT5G43360(AtPHT1;3), AT2G38940(AtPHT1;4), AT2G32830(PHT1;5), AT5G43340(AtPHT1;6), AT3G54700(AtPHT1;7), AT1G20860(AtPHT1;8), AT1G76430(AtPHT1;9), AT3G26570(AtPHT2;1), AT5G14040(AtPHT3;1), AT3G48850(AtPHT3;2), AT2G17270(AtPHT3;3), AT2G29650(AtPHT4;1), AT2G38060(AtPHT4;2), AT3G46980(AtPHT4;3), AT4G00370(PHT4;4), AT5G20380(AtPHT4;5), AT5G44370(AtPHT4;6), AT1G63010(AtPHT5;1), AT4G11810(PHT5;2), AT4G22990(AtPHT5;3),

To determine the evolutionary relationships of the members of the GmPHTs gene family genes with those of the soybean species, we constructed a phylogenetic tree comprising 92 PHTs proteins, from soybean (57), *Arabidopsis* (35), based on a multialignment via MEGA 6. Our results showed that all the PHTs homologs could be classified into six clades. GmPHT1 subfamily (14 members), GmPHT2 subfamily (2 members), GmPHT3 subfamily (9 members), GmPHT4 subfamily (12 members), GmPHT5 subfamily (6 members) and GmPHO1 subfamily (14 members), respectively, highly similar to each other. In addition, almost all of the soybean *PHTs* genes appeared as pairs with the PHTs members in *Arabidopsis* in terms of phylogenetic relationships (Fig. 1).

### Collinearity analysis among members of the GmPHTs gene family

**Figure 2:**
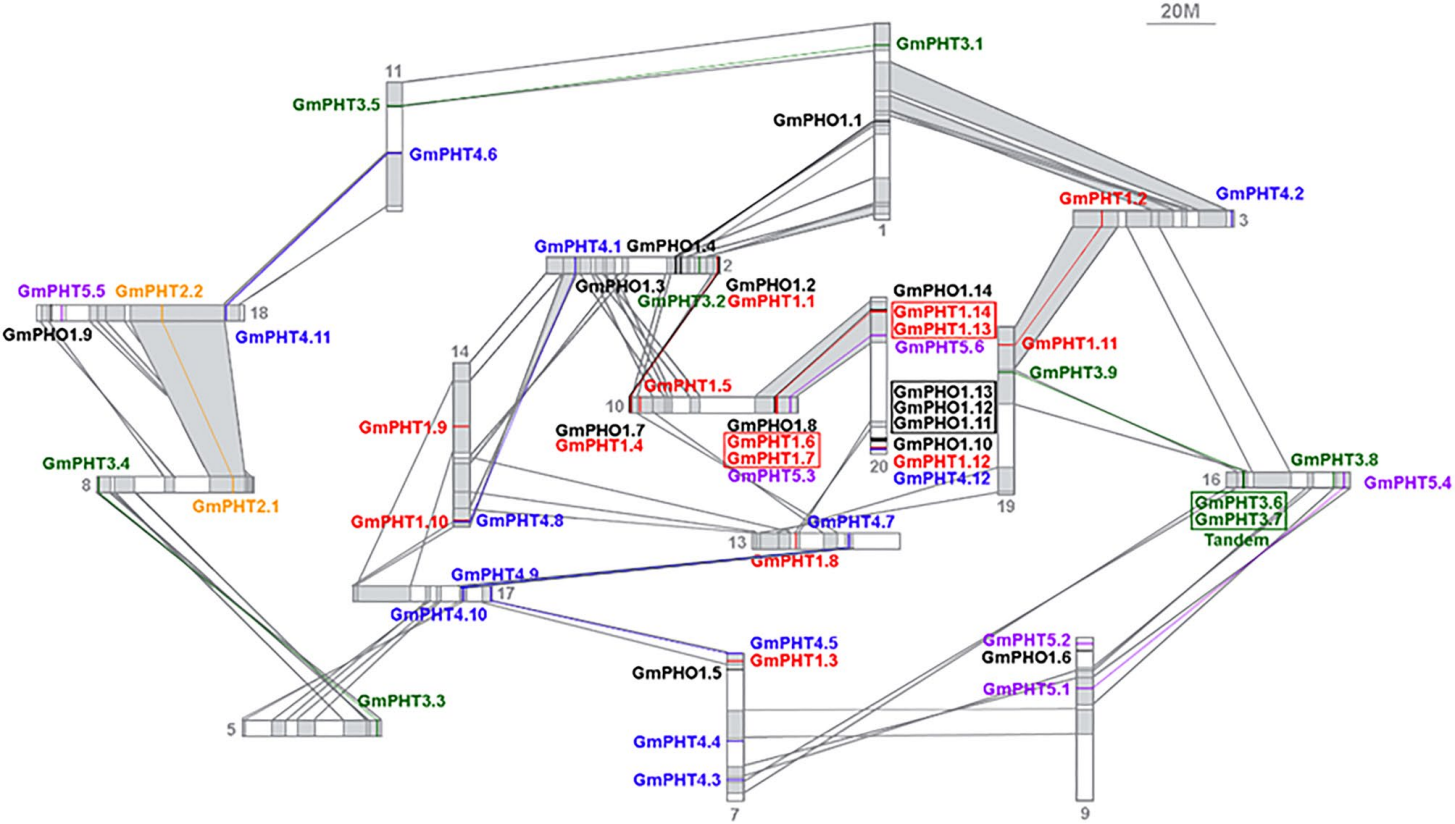
*GmPHTs* collinearity analysis results. *Note*: Members of the same subfamily are represented by the same color. Solid lines between genes indicate collinearity between the two. The genes enclosed in boxes are formed in the form of tandem repeats. The covariance data between chromosomes are cited Liu, et al. 2015^38^.

There are currently three ways in which duplicated genes are generated, including whole-genome duplication (polyploidy), large segmental duplication events and tandem duplication event^37^. Chromosomal distribution and Collinearity analysis of the *GmPHTs* gene (Fig. 2) shows that, 49% of the 57 *GmPHTs* gene family members were found to arise through large segmental duplication events and 14% through tandem duplication event. In the *GmPHT1* subfamily, the *GmPHT1.1* and *GmPHT1.4*, *GmPHT1.2* and *GmPHT1.11* genes are generated by a large segmental duplication event in the soybean chromosome; the *GmPHT1.6* and *GmPHT1.7*, *GmPHT1.13* and *GmPHT1.14* genes are generated by tandem duplication event, two of the GmPHT2 members were formed by large segmental duplication events. *GmPHT 3.1* and *GmPHT3.5*, *GmPHT3.3* and *GmPHT3.4*, and *GmPHT3.9* in the *GmPHT3* subfamily have common ancestor genes with *GmPHT3.6* and *GmPHT3.7* which were generated by large segmental duplication events; ancestor genes of *GmPHT3.6* and *GmPHT3.7* genes Tandem duplications occurred. Both the *GmPHT4* and *GmPHT5* subfamilies expanded the gene family size with only large segmental duplication events (*GmPHT4.1* and *GmPHT4.8*, *GmPHT4.5* and *GmPHT4.9*, *GmPHT4.6* and *GmPHT4.11* genes; *GmPHT5.1* and *GmPHT5:4*, *GmPHT5.3* and *GmPHT5.6* genes). of the *GmPHO1* subfamily, *GmPHO1.1* and *GmPHO1.4*, *GmPHO1.2* and *GmPHO1.7*, *GmPHO1.8* and *GmPHO1.14* genes were generated by large segmental duplication events, and *GmPHO1.11*, *GmPHO1.12* and *GmPHO1.13* genes were formed by tandem duplication event. It suggested that large segmental duplication also played a major driving force for GmPHTs evolution in addition to tandem duplication.

### Gene structure and protein-specific motif analysis of the GmPHTs gene

**Figure 3:**
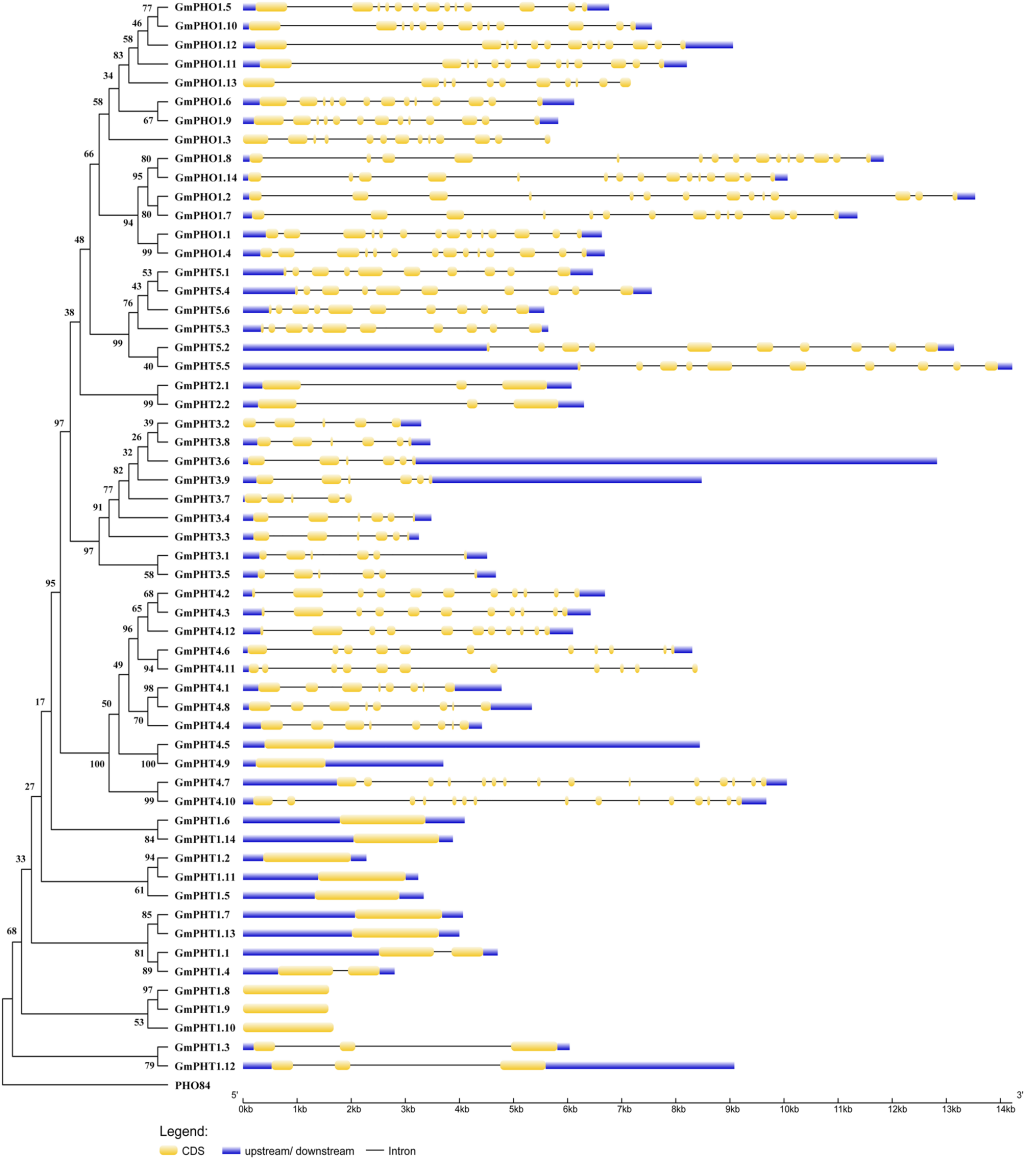
Analysis of the gene structure and protein-specific motifs of GmPHTs.

From the perspective of gene structure, the GmPHO1 subfamily can be divided into 2 Groups, and Group I contain 8 genes including *GmPHO1.3, GmPHO1.5, GmPHO1.6, GmPHO1.9, GmPHO1.10, GmPHO1.11, GmPHO1.12 and GmPHO1.13*. The three pairs of *GmPHO1* genes form Group II, which differs from the Group I genes mainly in the length of the first exon. Members of the *GmPHT3* subfamily have 5 or 6 exons, but the difference is mainly in whether the last exon is split or not. GmPHT4.1 and GmPHT4.8 both contain 8 exons, *GmPHT4.5* and *GmPHT4.9* genes contain only 1 exon, and *GmPHT4.7* and *GmPHT4.10* genes contain a maximum of 15 exons. The 14 members of the GmPHT1 subfamily can also be divided into two categories based on the results of gene structure, with the exception of *GmPHT1.1* and *GmPHT1.4*, which have two exons, the other 10 genes in Group III have only one exon, and *GmPHT1.3* and *GmPHT1.12*, which have similar structures and three exons. *GmPHO1* subfamily has a maximum of 10 specific motifs and the number of motifs is the same for each member of the *GmPHT3* and *GmPHT5* subfamilies. The protein sequences of the *GmPHT1* subfamily members contain 7–9 specific motifs, notably, the four gene pairs generated by large block repeats or tandem repeats and the *GmPHT1.5* gene have exactly the same 9 motifs, whereas the protein encoded by the five genes generated by non-repetitive events has only 7 motifs except for 8 and 9. Only the number of specific motifs varied significantly among members of the *GmPHT4* subfamily, ranging from 1 to 4 (Fig. 3).

### Identification of the cis-acting regulatory elements

**Figure 4:**
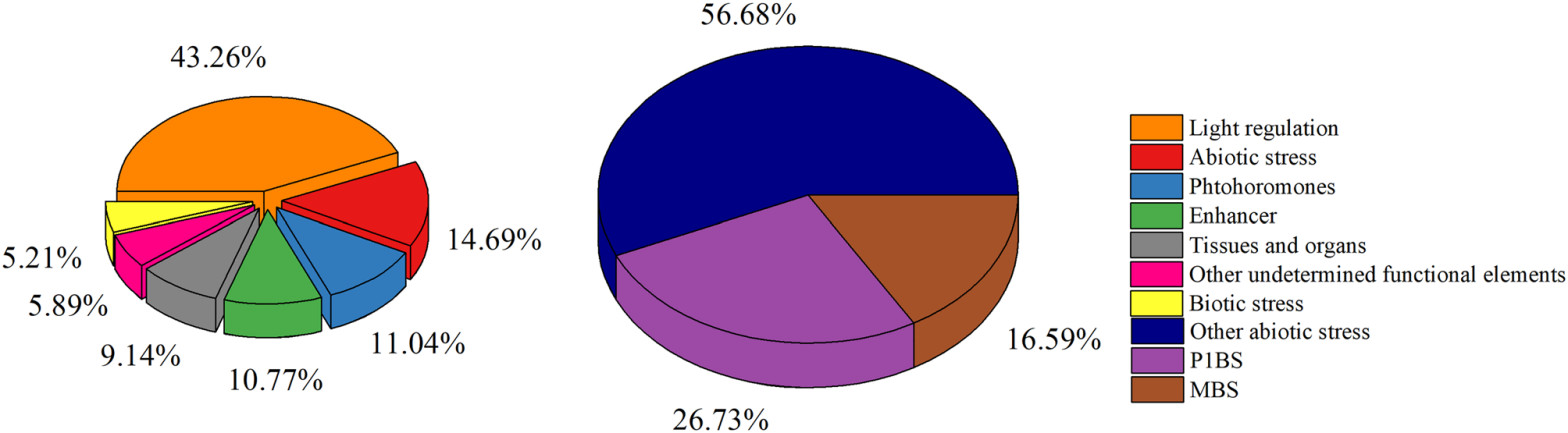
The promoter element composition of *GmPHTs* gene.

To understand the potential transcriptional regulation of GmPHTs, we performed the analysis based on the DNA sequences of the promoter regions. The 2.0-kb upstream region of the initiation codon as the promoter region of the gene to analyze which specific regulatory elements are present in the promoter region of each gene (Fig. 4, Table S2). The *GmPHTs* gene family has the most light-responsive elements, accounting for 43.26% of all promoter motifs and every gene has more or less of this type of promoter element. The next most abundant is the abiotic stress response element, which mainly responds to adverse environmental conditions such as heat, low temperature, anaerobic, drought, and low phosphorus. The phytohormone response elements mainly include abscisic acid, methyl jasmonate, gibberellin, ethylene, salicylic acid, and growth hormone response elements. Except for the *GmPHT3.1* gene, the promoters of all genes had phytohormone-like response elements, differing only in type and number. Biological stress response elements were the least, accounting for only about 5%, and none of the members of the PHT2 subfamily had biological stress response elements. It is thus hypothesized that each member of the *GmPHT* genes family has an important role in the growth and development of soybean and resistance to various external growth-adverse stimuli. The basic biological function of PHT proteins has been reported to be the transport of inorganic phosphorus, and the application of phosphorus has a mitigating effect on drought^39^, so among the promoter elements, the low phosphorus response element P1BS and the drought-inducing element MBS are the focus of our attention. Only 13 of the 57 genes have promoter regions containing both the low phosphorus response element P1BS and the drought-inducing element MBS. Therefore, further expression analysis was performed for 13 genes, including *GmPHT1.1, GmPHT1.7, GmPHT1.10, GmPHT1.14, GmPHT3.4, GmPHT3.5, GmPHT3.6, GmPHT4.7, GmPHT4.8, GmPHT4.10, GmPHO1.4, GmPHO1.5* and *GmPHO1.7*.

### Differential expression profiles of the GmPHTs genes under low phosphorus and drought stress

**Figure 5:**
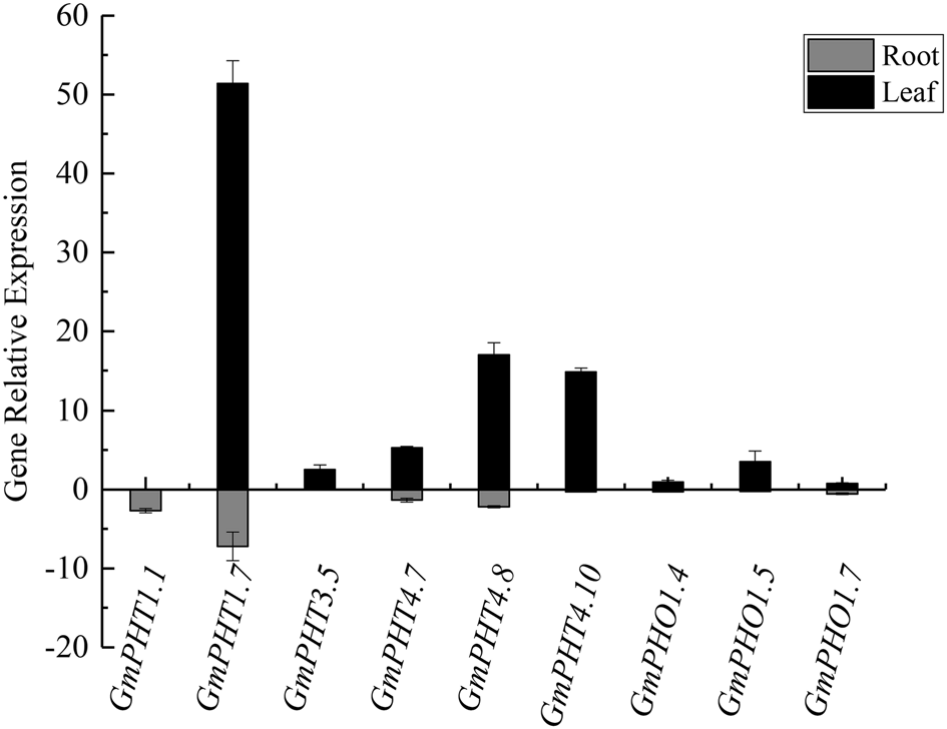
qRT-PCR results of GmPHT genes in soybean roots and leaves.

So far, little is known about the specificity of tissues or organs of the soybean PHT genes, which may elucidate their functions in detail, because the GmPHTs family members in soybean have not been systemically examined. By the results of the preliminary qRT-PCR experiments (Data not shown) and the gene microarray data of soybean, *GmPHT1.10, GmPHT1.14, GmPHT3.4* and *GmPHT3.6* genes were not expressed among the 13 genes. Therefore, only the remaining nine genes were examined separately for their expression under simultaneous low phosphorus drought treatment conditions (Fig. 5). *GmPHT1.1* gene was highly expressed in roots only, GmPHT3.5 gene was expressed in leaves only, *GmPHT1.7, GmPHT4.7, GmPHT4.8, GmPHT4.10, GmPHO1.4, GmPHO1.5* and *GmPHO1.7* genes were expressed in both roots and leaves. The GmPHT1.7 and *GmPHT4.8* genes were expressed in both leaves and roots, and it was inferred that the *GmPHT1.7* and *GmPHT4.8* genes may play important roles in both roots and leaves, and the *GmPHT1.1* gene acts synergistically in the roots during the seedling stage of soybean. In contrast, *GmPHT4.10, GmPHO1.4, GmPHO1.5,* and *GmPHO1.7* genes were expressed in the roots but at low levels, and these three genes may not play a major role in the roots.

**Figure 6:**
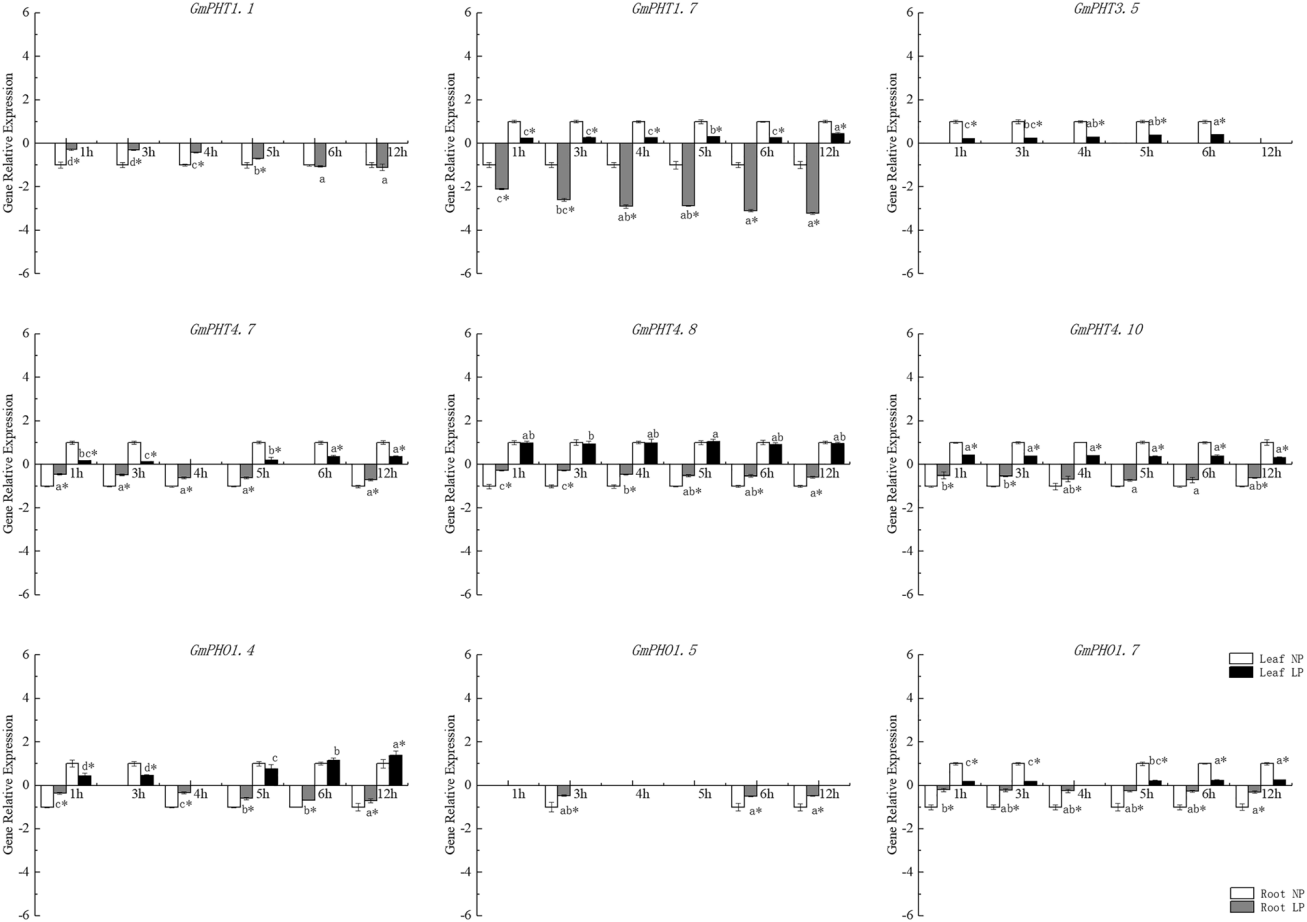
qRT-PCR results of nine GmPHT genes in roots and leaves of soybean treated with low phosphorus as well as drought.

The expression pattern of these nine genes changed again when soybean was treated with both low phosphorus and drought for 12 h (Fig. 6). *GmPHT3.5, GmPHT4.7, GmPHT4.10, GmPHO1.5, GmPHO1.7,* and *GmPHT1.7* was significantly reduced. The expression of the *GmPHT1.1* gene in the roots increased gradually with time, and its expression increased significantly with low phosphorus and drought treatment for 12 h. The expression pattern of *GmPHO1.4* gene in leaves was similar to that of *GmPHT1.1* gene, both of which decreased first and then showed a significant increase with time. Only the expression of *GmPHT4.8* gene in leaves was not affected by low phosphorus and drought, and the expression was basically the same as that of the control, but the expression in roots was significantly lower.

### Coexpression networks of the PHTs family genes in soybean

**Figure 7:**
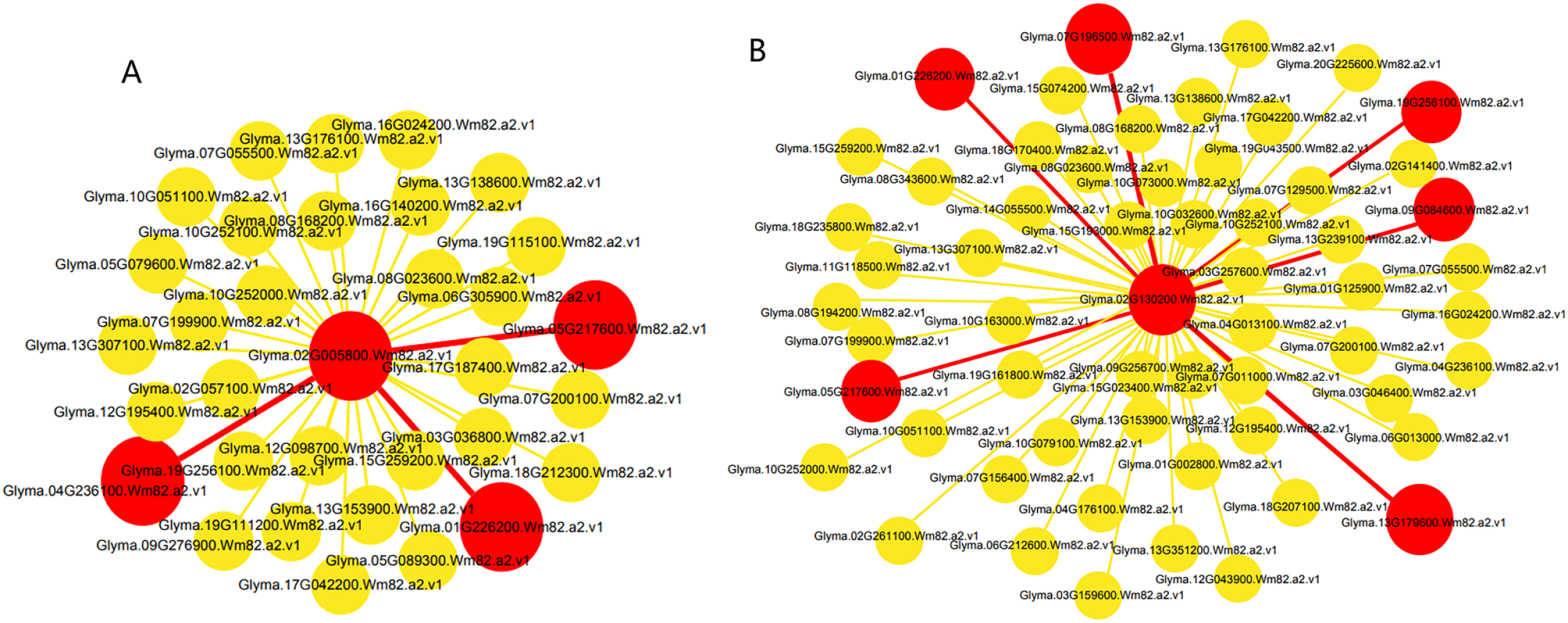
Coexpression networks of 2 PHTs family genes in soybean. (**A**), Glyma02G005800 (GmPHT1.1) coexpression network diagram; (**B**), Glyma02G130200 (GmPHO1.4) coexpression network diagram; The size of the node is proportional to the degree of the node, the more edges connected to the node, the greater its degree and the larger the node; the color indicates the strength of the interaction, the redder the color the stronger the interaction. The *PHTs* family genes located in the center of the network, while the most coexpressed genes were displayed in each network.

To further unravel the coexpression relationships between PHTs family genes and other genes, we calculated the interaction weight values of the target gene sets based on the FPKM values from the RNA-seq data. Figure 7 shows all the genes interacting with *GmPHTs* genes. In general, among the interacting genes, *GmPHT1:1* interacted with 31 genes, while *GmPHO1:4* interacted with 57 genes. Three genes interacted most strongly with *GmPHT1:1,* and six genes were co-expressed with *GmPHO1:4.* No other genes were found that interacted strongly with other *PHTs* genes under low phosphorus stress (Fig. 7). These include ubiquitin family genes, and ubiquitin-conjugating enzyme genes. Ubiquitination modifications play a central regulatory role in the plant response to low phosphorus stress and may be involved in regulating the uptake of phosphorus.

## Discussion

As an essential, nonsubstitutable element for plant growth, P plays a wide range of structural and biological roles^40^. P deficiency influences numerous biological processes in crop plants, such as rice, soybean and wheat, markedly reducing crop yield^41–44^. Plants need to obtain the phosphorus they need from the soil, but as the phosphorus concentration in the soil is lower than that in plant tissue, this sharp concentration gradient between the plant and the soil indicates the indispensable roles of PHTs, which can regulate Pi absorption ^45^.

Pi transporters currently identified in *Arabidopsis*, rice, soybean, maize, wheat, poplar, *Medicago truncatula*, and the *Halophyte E. salsugineum*^46^. The concerted action of Pi transporters ensures Pi acquisition and distribution among tissues and cytosolic Pi homeostasis^44^. Therefore, engineered alterations of the expression of Pi transporters provide an opportunity to optimize uptake and distribution of Pi in crops to improve yield^47^. A systematic analysis of the whole PHT gene family in soybean was conducted in this study, which will provide an opportunity to find candidate genes that play roles in phosphate use efficiency and abiotic stress response. A total of 57 PHT genes are identified in soybean and show quite high similarity to their corresponding members of the PHT gene family in *Arabidopsis* (Fig. 1). This implies that these PHT genes are evolutionarily conserved and functionally similar in soybean and *Arabidopsis*.

Gene duplication is considered one of the primary driving forces in the evolution of genomes and genetic systems. Of all the duplication patterns, segmental and tandem duplications have been purported to be the two main causes of gene family expansion in plants^48^. In the current research, 29 of the 57 *GmPHTs* (87.8%) in the soybean genome were associated with large segmental duplication events, Moreover, only 7 tandem duplication events were identified (Fig. 2). Taken together, our results indicate that large blockbuster duplication and tandem duplication events are the main force for the expansion of the PHT gene family in soybean. A relatively large number of members within a family suggests successful expansion and rearrangement of the genome by extensive duplication that occurred frequently during evolution^49^.

Cis elements may control the efficiency of promoters and thus regulate the expression of the genes that they control by interacting with the corresponding trans-regulatory factors^50^. Studies of cis elements might play a crucial role in dissecting the functions of genes. But the cis elements of all PHT gene family members in soybean have not yet been identified. In this study, the upstream regions of all the GmPHTs contain cis elements affecting light, hormone, defense and tissue-specific expression. These findings suggest that the expression of these *GmPHT* genes might be regulated by many factors, such as light-, hormone-, and defense-related factors. The presence of these cis elements in soybean *GmPHT* genes and their likely roles in regulating gene expression suggest that *GmPHT* genes may be involved in various stress responses in soybean Some cis elements function under phosphate deficiency, for example, the P1BS element can be bound to PHR1 (a MYB transcription factor) to potentially regulate the phosphate starvation response in *Arabidopsis*^51^, PHR1 has been suggested as a central integrator to role in the transcriptional regulation of phosphate starvation responses^52^. In the current study, P1BS-like elements were found in 13 of the 57 GmPHT promoters examined suggesting that expression of these genes might be influenced by Pi starvation. Similarly, P1BS-like elements have also been detected in *OsPHT* genes in rice^50^. These elements are found to be in the promoters of phosphate regulated genes, not only in *Arabidopsis* but also in other plant species such as barley and rice ^50,53,54^, which suggests that they may participate in a conserved signaling pathway for the phosphate starvation response in plants. The existence of P1BS-like elements in soybean, rice, and *Arabidopsis* suggests that they may serve a similar role in different species.

Drought and Pi deficiency stress often interfere with plant metabolism via various stress signals and hormonal changes that play essential roles in regulation processes under natural conditions^55^. So far, several studies have found that Pi transporters are involved in response to drought stress. Recently, several genes encoding Pi transporters from poplar were found to be regulated by drought stress, these genes especially those up-regulated by drought stress at low Pi level may contribute to drought tolerance of poplar plants in Pi-limited soils^11^^.^ Under drought stress, 18 *PHT* genes were upregulated in the leaves, and 11 *PHT* genes were downregulated in Brassica napus roots^56^. The expression of 15 *MdPHT* genes was upregulated in apple under drought stress^57^. Cao et al. (2020) found that overexpression of *StPHT1;7* in potato influenced plant growth and tolerance to drought stress^58^. Therefore, it is urgent that researchers develop plants with enhanced efficiency of soil phosphorus use under such conditions. Secondly, using drought inducible promoters to control the expression of Pi transporters or their regulatory factors may be a potential strategy to improve crop PUE under drought stress conditions. Drought can prevent Pi uptake by reducing Pi distribution in the root system^59^. Pi can also increase tissue osmotic pressure and enhance tolerance to drought stress by adjusting the water content in the cell structure and soluble sugar content in the cells^60^. Plants fertilised with Pi show moderate growth under drought compared to unfertilised plants^61^. In addition, phosphorus fertilizers are often used to reduce Pi deficiency in the soil, improve drought tolerance and promote plant growth^62^. In Alnus cremastogyne, phosphorus fertilisation alleviates drought stress by regulating its antioxidant and osmotic potential^63^. Several recent studies have highlighted the role of phosphorus in alleviating drought stress in plants by promoting growth, especially of seedlings^64^. Plants primarily use phosphate transporters (PHT) located in the cell membranes of plant roots to take up Pi from the soil, which is then transported into the plant^65^. Increased phosphorus uptake enhances tolerance to low phosphorus^66,67^ and drought stress^62,68^. Therefore, engineered alterations of the expression of Pi transporters provide an opportunity to optimize uptake and distribution of Pi in crops to improve yield.

The number of Pi transporters present may reflect the complexity and significance of the process of Pi transport^5^. Most PHT1 family genes expressed in roots show up-regulation in phosphate deprived *Arabidopsis* plants; In rice, high-affinity of OsPHT9 and OsPHT10 for Pi transport have been reported, as well as specific induction of their expression by Pi starvation^69^ The transcript levels of a few PHT genes in *Arabidopsis* were increased by Pi deficiency^70^; however, the expression of most PHO family members was not induced by Pi deficiency in poplar, suggesting that PHT genes might have different functions in woody species and herbs. In addition, some PHT4 family members might be low-affinity transporters, consistent with the relatively high Pi levels that have been reported in the cytosol and other subcellular compartments^7^. In this study, the expression patterns of *GmPHT* genes were analyzed under low phosphorus, drought and simultaneous low phosphorus drought stress conditions, and the results showed that the expression patterns of the genes differed under different treatment conditions. These results suggest that of crosstalk occurs among the P-starvation response and salt and drought stress responses, and Pi uptake in plants likely changes in association with the altered expression of the PHT1 genes under drought and salt stresses^47^.Our results show that under simultaneous drought and low phosphorus treatments, the expression of *GmPHT1.7* and *GmPHT1.1* genes in roots significantly increased, while the expression of *GmPHO1.4* gene in leaves showed a significant increase with the continuation of treatment time, and the expression of this gene in roots significantly decreased. The expression of GmPHT4.8 gene in leaves was not affected by low phosphorus and drought, but the expression in roots was significantly reduced (Fig. 6). The identification of these genes is therefore of great significance for the development of transgenic crops to cope with low phosphorus and drought stress.

## Conclusions

Plant Pi transporters play an essential role in Pi acquisition and distribution. The family of phosphate transporters (PHTs) mediates the uptake and translocation of Pi inside the plants. In this study, a total of 57 PHT-domain-containing protein genes were identified in soybean genome, (GmPHT1, GmPHT2, GmPHT3, GmPHT4, GmPHT5 and GmPHO1) had been found. Only 13 of the 57 genes have promoter regions containing both the low phosphorus response element P1BS and the drought-inducing element MBS, 4 genes were not expressed among the 13 genes. Therefore, only the remaining nine genes were examined separately for their expression under simultaneous low phosphorus drought treatment conditions. *GmPHT1.7, GmPHT1.1, GmPHO1.4* and *GmPHT4.8* genes displayed various expression patterns under low phosphorus and drought stress. The above results implied that they may play different roles in phosphate nutrition of Soybean different tissues and development stages. These results provide references for the further study of Soybean PHT-domain-containing proteins family genes. Moreover, this study provides the selection of candidate genes for functional research and genome editing in Soybean phosphate nutrition.

## Materials and methods

### Identification of the GmPHT family genes in soybean

The Arabidopsis (*Arabidopsis thaliana*) PHT protein sequence was used for TBlastN homology alignment in the soybean genome database (Phytozome V12.1) to obtain sequences similar to members of the *AtPHT* gene family. The obtained sequences were then analyzed in the NCBI CDD database (v3.15) for conserved structural domains to identify whether the obtained similar sequences were GmPHTs proteins. The sequence of the protein finally identified as GmPHTs was sequence corrected in the NCBI soybean database. Data on the isoelectric point, molecular weight, and hydrophilicity of the proteins were obtained using the ProtParam tool in the ExPASy (https://web.expasy.org/) online analysis website.

### Phylogenetic, Multiple alignment and protein motif analysis

The PHT protein sequences of *Arabidopsis* and soybean were selected for multiple sequence alignment using the ClustalW program in BioEdit (v7.0.5) software. The PHT protein sequences of yeast were used as outgroup to construct a phylogenetic tree by MEGA (v7.0.26) software. The method used was the Jones-Taylor-Thornton (JTT) model, and Bootstrap was 1000 replicates.

The PLAZA website (https://bioinformatics.psb.ugent.be/plaza) was used to analyze the collinearity of the soybean genome and finally to obtain the regions where large segments of duplication occurred in the soybean genome.

The number of introns and exons of genes was analyzed and counted using the online analysis website GSDS (v2.0) (http: //gsds.cbi.pku.edu.cn). The unique or shared motifs in the proteins were done using the MEME line analysis website (http://meme-suite.org/).

### Promoter element analysis in GmPHT genes

The gene sequence was obtained from the soybean genome database phytozome (V12.1) and the 2000 bp sequence upstream of the gene was used as the promoter region. The promoter element prediction was performed for all *GmPHT* members using the promoter online prediction website PlantCare (http://bioinformatics.psb.ugent.be/ webtools/plantcare/html/). The promoter elements other than the basic promoter elements (TATA box and CAAT box) were mapped using the GSDS online mapping tool for promoter elements.

### Plant materials and growth condition

The experiments were conducted with the main cultivar Changnong 26 in Jilin Province (Validation No.: JI Audited Bean 2,010,004). To identify tissue-preferentially expressed genes, seedlings of Chang Nong 26 were grown under normal conditions and cultured in sand culture at 25 °C with 50% humidity and 13 h of light and 9 h of darkness, using 1/4 Hoagland (containing 0.5 mM KH_2_PO_4_) as the culture medium to provide essential nutrients for the soybean. The plants were converted to hydroponic culture when they reached the three-leaf stage, and the culture medium was still 1/4 Hoagland containing 0.5 mM KH_2_PO_4_. After 12 h of hydroponic culture, the plants were transferred to 1/4 Hoagland culture with 0.01 mM KH_2_PO_4_, 1/4 Hoagland culture with 2% PEG8000 (containing 0.5 mM KH_2_PO_4_) and 1/4 Hoagland culture with 2% PEG8000 containing 0.01 mM KH_2_PO_4_ for Hydroponic culture. The plants were cultivated in a glasshouse (natural light, 75% relative humidity) and irrigated with 50 ml nutrient solution. Three treatments were set up with three replications, continuous normal phosphorus treatment, continuous low phosphorus, and stressed low phosphorus treatment. Samples were taken on time for 12 h in hydroponics, and the roots and leaves were stored separately at − 80 °C for backup.

### Coexpression networks of the GmPHT family genes using RNA-seq data

Gene coexpression network analysis was performed based on the RNA-seq data. For the RNA-seq experiment, fully expanded leaves and roots were taken when the plants were treated with low phosphorus nutrient solution until to 3 leaves separately for RNA extraction with three biological replicates. The transcript abundance (FPKM value) of each gene was calculated based on the length of the gene and the reads mapped to that gene. The interactions of the target gene sets were retrieved from the STRING protein database (http://string-db.org/), and the weight value of the target gene sets was calculated using the WGCNA R package based on the FPKM values. The gene coexpression networks were visualized by Cytoscape software^71^.

### Quantitative real-time PCR analysis

RNA was extracted using Trizol (TAKARA, Beijing) and reverse transcribed using PrimeScriptTM RT reagent Kit with gDNA Eraser kit (TAKARA, Beijing). Fluorescent quantitative PCR primers were designed according to the specific sequences of each gene, see Table S1. Letin was used as the internal reference gene, and the fluorescent quantitative PCR was performed using the TAKARA SYBR Premix Ex Taq II kit (TAKARA, Beijing, China) with an ABI StepOne Plus real-time fluorescent quantitative PCR instrument (ABI, USA, model 7300). 25 μl of the reaction system contained 1 μl cDNA, 10 μl SYBR The PCR program was: 95 °C for 10 min (pre-denaturation), 95 °C for 3 s (denaturation) and 60 °C for 1 min (annealing-extension) for 40 cycles. The lysis curve procedure was: 95 °C for 15 s, 60 °C for 1 min, and 95 °C for 15 s. Data were calculated using the 2^−ΔΔct^ algorithm for relative expression^72^. All experiments were performed in three replicates. Statistix (v 8.1) software was used to analyze the significance of the data.

### Statistical analysis of data

Statistics was performed by Duncan’s test or Student’s t test. Significance of differences was defined as *P < 0.05, **P < 0.01.

## Supplementary Information

Supplementary Information.

## Data Availability

The original contributions presented in the study are included in the article/Supplementary Material. The Supplementary Material for this article can be found online at: https://www.ncbi.nlm.nih.gov/sra/PRJNA871448. Accession number: PRJNA871448, NCBI SRA database.

## References

[1] Roch GV, Maharajan T, Ceasar SA, Ignacimuthu S (2019). The role of PHT1 family transporters in the acquisition and redistribution of phosphorus in plants. CRC. Crit. Rev. Plant Sci..

[2] Razaq M, Zhang P, Shen H, Salahuddin S (2017). Influence of nitrogen and phosphorous on the growth and root morphology of Acer mono. PLoS ONE.

[3] Hui Y, Dong L (2010). Signaling components involved in plant responses to phosphate starvation. J. Chin. Bot. (Engl. Vers.).

[4] Raghothama KG (2000). Phosphate transport and signaling. Curr. Opin. Plant Biol..

[5] Tingting S, Mingjun Li, Yun S, Lingyan Yu, Fengwang Ma (2017). Comprehensive genomic identification and expression analysis of the phosphate transporter (PHT) gene family in apple. J. Front. Plant Sci..

[6] Schachtman DP, Reid RJ, Ayling S (1998). Phosphorus uptake by plants: From soil to cell. Plant Physiol..

[7] Mimura T (1999). Regulation of phosphate transport and homeostasis in plant cells. Int. Rev. Cytol..

[8] Rausch C, Bucher M (2002). Molecular mechanisms of phosphate transport in plants. Planta.

[9] Guo B, Jin Y, Wussler C, Blancaflor EB, Motes CM, Versaw WK (2008). Functional analysis of the Arabidopsis PHT4 family of intracellular phosphate transporters. New Phytol..

[10] Liu TY, Huang TK, Yang SY, Hong YT, Huang SM, Wang FN, Chiang SF, Tsai SY, Lu WC, Chiou TJ (2016). Identification of plant vacuolar transporters mediating phosphate storage. Nat. Commun..

[11] Zhang C, Meng S, Li M, Zhao Z (2016). Genomic identification and expression analysis of the phosphate transporter gene family in poplar. Front. Plant Sci..

[12] Ceasar SA, Baker A, Ignacimuthu S (2017). Functional characterization of the PHT1 family transporters of foxtail millet with a novel Agrobacterium-mediated transformation procedure. J. Sci. Rep..

[13] Li RL, Wang JL, Xu L, Sun MH, Zhao HY (2020). Functional analysis of phosphate transporter OsPHT4 family members in rice. J. Rice Sci..

[14] Muchhal US, Pardo JM, Raghothama KG (1996). Phosphate transporters from the higher plant Arabidopsis thaliana. Proc. Natl. Acad. Sci. U. S. A..

[15] Lopez-Arredondo DL, Leyva-González MA, González-Morales SI, López-Bucio J, Herrera-Estrella L (2014). Phosphate nutrition: Improving low-phosphate tolerance in crops. Plant Biol..

[16] Mudge SR, Rae AL, Diatloff E, Smith FW (2002). Expression analysis suggests novel roles for members of the Pht1 family of phosphate transporters in Arabidopsis. Plant J..

[17] Wang H, Xu Q, Kong YH, Chen Y, Duan JY, Wu WH, Chen YF (2014). Arabidopsis WRKY45 transcription factor activates PHOSPHATE TRANSPORTER1; 1 expression in response to phosphate starvation. Plant Physiol..

[18] Shi SL, Wang DF, Yan Y, Zhang F, Wang HD, Sun SB, Xu GH (2013). Function of phosphate transporter OsPHT2; 1 in improving phosphate utilization in rice. Chin. J. Rice Sci..

[19] Kiiskinen M, Korhonen M, Kangasjärvi J (1997). Isolation and characterization of cDNA for a plant mitochondrial phosphate translocator (Mpt1): Ozone stress induces Mpt1 mRNA accumulation in birch (Betula pendula Roth). J. Plant Mol. Biol..

[20] Nakamori K, Takabatake R, Umehara Y, Kouchi H, Izui K, Hata S (2002). Cloning, functional expression, and mutational analysis of a cDNA for lotus Japonicus mitochondrial phosphate transporter. J. Plant Cell Physiol..

[21] Miyaji T, Kuromori T, Takeuchi Y, Yamaji N, Yokosho K, Shimazawa A, Sugimoto E, Omote H, Ma JF, Shinozaki K, Moriyama Y (2015). AtPHT4; 4 is a chloroplast-localized ascorbate transporter in Arabidopsis. Nat. Commun..

[22] Irigoyen S, Karlsson PM, Kuruvilla J, Spetea C, Versaw WK (2011). The sink-specific plastidic phosphate transporter PHT4; 2 influences starch accumulation and leaf size in Arabidopsis. Plant Physiol..

[23] Wang GY, Shi JL, Ng G, Battle SL, Zhang C, Lu H (2011). Circadian clock-regulated phosphate transporter PHT4; 1 plays an important role in Arabidopsis defense. Mol. Plant..

[24] Wang C, Yue W, Ying Y, Wang S, Secco D, Liu Y, Whelan J, Tyerman SD, Shou H (2015). OsSPX-MFS3, a vacuolar phosphate efflux transporter, is involved in maintaining Pi homeostasis in rice. Plant Physiol..

[25] Qin L, Zhao J, Tian J, Chen L, Sun Z, Guo Y, Lu X, Gu M, Xu G, Liao H (2012). The high-affinity phosphate transporter GmPT5 regulates phosphate transport to nodules and nodulation in soybean. J. Plant Physiol..

[26] Wang X, Yan X, Liao H (2010). Genetic improvement for phosphorus efficiency in soybean: A radical approach. Ann. Bot..

[27] He J, Jin Y, Du YL, Wang T, Turner NC, Yang RP, Siddique KHM, Li FM (2017). Genotypic variation in yield, yield components, root morphology and architecture, in soybean in relation to water and phosphorus supply. Front. Plant Sci..

[28] Gutierrez-Boem FH, Thomas GW (1999). Phosphorus nutrition and water deficits infield-grown soybeans. Plant Soil.

[29] Jin J, Wang GH, Liu XB, Pan XW, Herbert SJ, Tang CX (2006). Interaction between phosphorus nutrition and drought on grain yield, and assimilation of phosphorus and nitrogen in two soybean cultivars differing in protein concentration in grains. J. Plant Nutr..

[30] Ogoke IJ, Carsky RJ, Togun AO, Dashiell K (2003). Effect of P fertilizer application on N balance of soybean crop in the guinea savanna of Nigeria. Agr. Ecosyst. Environ..

[31] Rotaru V, Sinclair TR (2009). Interactive influence of phosphorus and iron on nitrogen fixation by soybean. Environ. Exp. Bot..

[32] Sinclair TR, Serraj R (1995). Legume nitrogen-fixation and drought. Nature.

[33] Serraj R, Sinclair TR, Purcell LC (1999). Symbiotic N2fixation response to drought. J. Exp. Bot..

[34] Zheng HF, Chen LD, Yu XY, Zhao XF, Ma Y, Ren ZB (2015). Phosphorus control as an effective strategy to adapt soybean to drought at the reproductive stage: Evidence from field experiments across northeast China. J. Soil Use Manag..

[35] Basal O, Szabó A (2020). Physiomorphology of soybean as affected by drought stress and nitrogen application. Scientifica (Cairo)..

[36] Feng YY, He J, Turner NC, Siddique Kadambot HM, Li FM (2021). Phosphorus supply increases internode length and leaf characteristics, and increases dry matter accumulation and seed yield in soybean under water deficit. J. Agron..

[37] Zhu Y, Wu N, Song W, Yin G, Qin Y, Yan Y, Hu Y (2014). Soybean (Glycine max) expansin gene superfamily origins: Segmental and tandem duplication events followed by divergent selection among subfamilies. J. Bmc Plant Biol..

[38] Liu HJ, Tang ZX, Han XM, Yang ZL, Zhang FM, Yang HL, Liu YJ, Zeng QY (2015). Divergence in enzymatic activities in the soybean GST supergene family provides new insight into the evolutionary dynamics of whole-genome duplicates. Mol. Biol. Evol..

[39] Sun T, Zhou B, Pei T, Meng H, Zhang J, Ma F, Wei Q (2021). Phosphate transporter MdPHT1;7 enhances phosphorus accumulation and improves low phosphorus and drought tolerance. J. Plant Biol..

[40] Hawkesford M, Horst W, Kichey T, Lambers H, Schjoerring J, Inge Skrumsager M, White P (2012). Chapter 6: Functions of macronutrients. Marschner’s mineral nutrition of higher plants.

[41] Gamuyao R, Chin JH, Pariasca-Tanaka J, Pesaresi P, Catausan S, Dalid C, Slamet-Loedin I, Tecson-Mendoza EM, Wissuwa M, Heuer S (2012). The protein kinase Pstol1 from traditional rice confers tolerance of phosphorus deficiency. Nature.

[42] Wu WW, Lin Y, Liu PD, Chen QQ, Tian J, Liang CY (2018). Association of extracellular dNTP utilization with a GmPAP1-like protein identified in cell wall proteomic analysis of soybean roots. J. Exp Bot..

[43] Lin Y, Chen G, Hu H, Yang X, Liu Y (2020). Phenotypic and genetic variation in phosphorus-deficiency-tolerance traits in Chinese wheat landraces. BMC Plant Biol..

[44] Remy E, Cabrito TR, Batista RA, Teixeira MC, Sá-Correia I, Duque P (2012). The Pht1;9 and Pht1;8 transporters mediate inorganic phosphate acquisition by the Arabidopsis thaliana root during phosphorus starvation. J. New Phytologist..

[45] Nussaume L (2011). Phosphate import in plants: Focus on the PHT1 transporters. Front. Plant Sci..

[46] Wang D, Lv S, Jiang P, Li Y (2017). Roles, regulation, and agricultural application of plant phosphate transporters. J. Front. Plant Sci..

[47] López-Arredondo DL, Leyva-González MA, González-Morales SI, López-Bucio J, Herrera-Estrella L (2014). Phosphate nutrition: Improving low-phosphate tolerance in crops. J. Annu. Rev. Plant Biol..

[48] Cannon SB, Mitra A, Baumgarten A, Young ND, May G (2004). The roles of segmental and tandem gene duplication in the evolution of large gene families in Arabidopsis thaliana. BMC Plant Biol..

[49] Liu F, Ghang XJ, Ye Y, Xie WB, Wu P, Lian XM (2011). Comprehensive sequence and whole-life-cycle expression profile analysis of the phosphate transporter gene family in rice. Mol Plant..

[50] Sobkowiak L, Bielewicz D, Malecka EM, Jakobsen I, Albrechtsen M, Szweykowska-Kulinska Z, Pacak A (2012). The role of the P1BS element containing promoter-driven genes in Pi transport and homeostasis in plants [J]. Front. Plant Sci..

[51] Rubio V, Linhares F, Solano R, Martín AC, Iglesias J, Leyva A, Paz-Ares J (2001). A conserved MYB transcription factor involved in phosphate starvation signaling both in vascular plants and in unicellular algae. Genes Dev..

[52] Regla B, Gabriel C, Francisco L, Isabel PM, Vicente R, Julian PP, Ecker JR (2010). A central regulatory system largely controls transcriptional activation and repression responses to phosphate starvation in Arabidopsis. PLoS Genet..

[53] Schünmann PH, Richardson AE, Smith FW, Delhaize E (2004). Characterization of promoter expression patterns derived from the Pht1 phosphate transporter genes of barley (Hordeum vulgare L.). J. Exp. Bot..

[54] Schünmann PH, Richardson AE, Vickers CE, Delhaize E (2004). Promoter analysis of the barley Pht1;1 phosphate transporter gene identifies regions controlling root expression and responsiveness to phosphate deprivation. Plant Physiol..

[55] Martin J, Saad S, Faouzi B, Olomide O, Abeer H, Fathi A, Phan LS (2017). Comparative analysis of the combined effects of different water and phosphate levels on growth and biological nitrogen fixation of nine cowpea varieties. J. Front. Plant Sci..

[56] Li Y, Wang X, Zhang H, Wang SL, Ye XS, Shi L, Xu FS, Ding GD (2019). Molecular identification of the phosphate transporter family 1 (PHT1) genes and their expression profiles in response to phosphorus deprivation and other abiotic stresses in Brassica napus. PLoS ONE.

[57] Sun T, Li M, Shao Y, Yu L, Ma F (2017). Comprehensive genomic identification and expression analysis of the phosphate transporter (PHT) gene family in apple. Front Plant Sci..

[58] Cao MX, Liu HZ, Zhang C, Wang DD, Liu XF, Chen Q (2020). Functional analysis of StPHT1;7, a Solanum tuberosum L. phosphate transporter gene, in growth and drought tolerance. Plants-Basel..

[59] Ackerson RC (1985). Osmoregulation in cotton in response to water stress: III effects of phosphorus fertility. Plant Physiol..

[60] Liu C, Rubæk GH, Liu F, Andersen MN (2015). Effect of partial root zone drying and deficit irrigation on nitrogen and phosphorus uptake in potato. Agric. Water Manag..

[61] Graciano C, Guiamét JJ, Goya JF (2005). Impact of nitrogen and phosphorus fertilization on drought responses in eucalyptus grandis seedlings. For Ecol. Manag..

[62] Faustino LI, Bulfe NM, Pinazo MA, Monteoliva SE, Graciano C (2013). Dry weight partitioning and hydraulic traits in young Pinus taeda trees fertilized with nitrogen and phosphorus in a subtropical area. Tree Physiol..

[63] Tariq A, Pan K, Olatunji OA, Graciano C, Zhang A (2018). Phosphorous fertilization alleviates drought effects on Alnus cremastogyne by regulating its antioxidant and osmotic potential. Sci. Rep..

[64] Tng DY, Janos DP, Jordan GJ, Weber E, Bowman DM (2014). Phosphorus limits eucalyptus grandis seedling growth in an unburnt rain forest soil. Front Plant Sci..

[65] Mimura T, Mitsuhashi N, Sekiguchi Y, Ohnishi M (2003). Phosphate transport and intracellular distribution in plants. Tanpakushitsu Kakusan Koso.

[66] Kakar KM, Muhammad T, Taj FH, Nawab K (2001). Phosphorus use efficiency of soybean as affected by phosphorus application and inoculation. J. Agron..

[67] Bucher M, Rausch C, Daram P (2015). Molecular and biochemical mechanisms of phosphorus uptake into plants. J. Plant Nutr. Soil Sci..

[68] Begum N, Ahanger MA, Zhang LX (2020). AMF inoculation and phosphorus supplementation alleviates drought induced growth and photosynthetic decline in Nicotiana tabacum by up regulating antioxidant metabolism and osmolyte accumulation. Environ. Exp. Bot..

[69] Wang X, Wang Y, Piñeros MA, Wang Z, Wang W, Li C, Wu Z, Kochian LV, Wu P (2014). Phosphate transporters OsPHT1;9 and OsPHT1;10 are involved in phosphate uptake in rice. Plant Cell Environ..

[70] Ribot C, Wang Y, Poirier Y (2008). Expression analyses of three members of the AtPHO1 family reveal differential interactions between signaling pathways involved in phosphate deficiency and the responses to auxin, cytokinin, and abscisic acid. Planta.

[71] Saito R, Smoot ME, Ono K, Ruscheinski J, Wang PL, Lotia S, Pico AR, Bader GD, Ideker T (2012). A travel guide to cytoscape plugins. Nat. Method..

[72] Livak KJ, Schmittgen TD (2001). Analysis of relative gene expression data using real-time quantitative PCR and the 2(-delta delta C (T)) method. Method.

