# Supplementary material for: Comprehensive sequence and expression profile analysis of the phosphate transporter gene family in soybean
Source: Sci Rep. 2022 Dec 3;12:20883. doi: 10.1038/s41598-022-25378-w (PMC9719489; doi:10.1038/s41598-022-25378-w)
Supplement: Supplementary file 1 — Supplementary Information. [file 41598_2022_25378_MOESM1_ESM.zip › Supplementary material/Table S1.docx]

Table S1 qRT-PCR primers for GmPHT genes

| Gene | Forward primer(5'-3') | Reverse primer(5'-3') |
| --- | --- | --- |
| *Letin* | TGGGACAGCGAAACCGGCAA | CCATCGTCGGATTGGGGCTGA |
| *GmPHT1.1* | CAGGTTCTGGCTAGGGTTTG | ACATAGTCAAATGCGGGGTC |
| *GmPHT1.7* | TGACCACAAGTACGATCTTCC | CGCCAATAGTAGGTAAGAGCA |
| *GmPHT3.5* | TAGTGCGTCTGCTGAAGTGTT | GGTAGAATCCTCGTGTGCCT |
| *GmPHT4.7* | AAATGCTAAAGAAACCCAGAG | GAAGAAGGAGGACTGAACCA |
| *GmPHT4.8* | ATCAAATACGGCTGGAACAT | AGTAGCGAATAGGCAATAGAAA |
| *GmPHT4.10* | GGATTCTCACAAGTGGTTTAGC | AGGAACAGCCCCAACAGTAT |
| *GmPHO1.4* | AAATACCTTAGACCGAGCCAA | AAATGTAGGAACACAAGGCTGA |
| *GmPHO1.5* | AACATCGTTTATCGGTCAAG | AAGTCTCCCCAACCATAGTAG |
| *GmPHO1.7* | GTGAACAGTCCAATGAAACCA | TCGGAACTAATGAAATGGGA |
