# Supplementary material for: Comprehensive sequence and expression profile analysis of the phosphate transporter gene family in soybean
Source: Sci Rep. 2022 Dec 3;12:20883. doi: 10.1038/s41598-022-25378-w (PMC9719489; doi:10.1038/s41598-022-25378-w)
Supplement: Supplementary file 1 — Supplementary Information. [file 41598_2022_25378_MOESM1_ESM.zip › Supplementary material/Table S2.docx]

Table S2 The promoter element composition of *GmPHTs* gene

| Name | PHO1 | PHT1 | PHT2 | PHT3 | PHT4 | PHT5 | Total number per category | total | ratio |
| --- | --- | --- | --- | --- | --- | --- | --- | --- | --- |
| Light regulation | 157 | 171 | 30 | 88 | 125 | 68 | 639 | 1477 | 0.432633717 |
| Phtohoromones | 42 | 41 | 4 | 23 | 39 | 14 | 163 | 1477 | 0.110358835 |
| Abiotic stress | 48 | 65 | 5 | 37 | 41 | 21 | 217 | 1477 | 0.146919431 |
| Biotic stress | 15 | 26 | 0 | 18 | 14 | 4 | 77 | 1477 | 0.052132701 |
| Tissues and organs | 34 | 27 | 9 | 20 | 27 | 18 | 135 | 1477 | 0.09140149 |
| Other undetermined functional elements | 31 | 17 | 2 | 12 | 16 | 9 | 87 | 1477 | 0.058903182 |
| P1BS | 10 | 27 | 0 | 4 | 7 | 10 | 58 | 217 | 0.267281106 |
| MBS | 11 | 6 | 0 | 5 | 14 | 0 | 36 | 217 | 0.165898618 |
| Other abiotic stress | 27 | 32 | 5 | 28 | 20 | 11 | 123 | 217 | 0.566820276 |
| Enhancer | 39 | 37 | 1 | 6 | 47 | 29 | 159 | 1477 | 0.107650643 |
